# Refining the APGAR Score Cutoff Values and Viability Classes According to Breed Body Size in Newborn Dogs

**DOI:** 10.3390/ani12131664

**Published:** 2022-06-28

**Authors:** Maria Cristina Veronesi, Massimo Faustini, Monica Probo, Alessandro Rota, Jasmine Fusi

**Affiliations:** 1 Department of Veterinary Medicine and Animal Sciences, Università degli Studi di Milano, Via dell’Università 6, 26900 Lodi (LO), Italy; maria.veronesi@unimi.it (M.C.V.); massimo.faustini@unimi.it (M.F.); jasmine.fusi@unimi.it (J.F.); 2 Practitioner, DVM, Ambulatorio Veterinario Associato Pellegrini e Rota, Via Ungaretti, 69, 24030 Almenno San Bartolomeo (BG), Italy; ale.vet@libero.it

**Keywords:** Apgar score, viability class, newborn dog, breed body size

## Abstract

**Simple Summary:**

The Apgar score, based on the easy and quick evaluation of some neonatal parameters, represents a key tool in the routine assessment of newborns in many species, dog included. Recently, a reevaluation of the Apgar score used in canine species has become necessary to verify possible adjustment and redefinition of cutoff and newborn viability classes, also according to the breed. With this aim, data from 234 dog litters born by caesarean section were retrospectively evaluated and grouped according to breed body size. According to results, new cutoff values and viability classes were refined and a narrower class of moderately distressed puppy was defined, especially for small-sized breeds; moreover, the small-sized puppies were the most represented in the severely distressed class, but had the best chance of survival in comparison to large-sized newborns. In conclusion, the refining of the Apgar score in dog species is imperative, and one must consider the differences related to breed body-size characteristics, with cutoff values and viability classifications adapted to the specific breed body size.

**Abstract:**

The Apgar score (AS) represents a key tool for neonate assessment, but the possible breed effect on AS in newborn puppies has never been investigated. Therefore, data from 234 dog litters born by caesarean section, grouped according to breed body size (BBS) (small, medium, large), were evaluated. Live-birth puppies were assessed through AS within 5 min of delivery, and classified in viability classes: 0–3 severely distressed, 4–6 moderately distressed, 7–10 not distressed. Statistical analysis evaluated possible differences of AS and viability class according to BBS, and between BBS and puppies’ mortality. Results showed no differences in the distribution of mortalities among BBSs. However, an effect of BBS on the AS was found, with small-sized puppies being the most represented in the severely distressed class, but having the best survival chance compared to large-sized newborns. Through receiver-operating-characteristics analysis, the AS new cutoff values for survival and for death <24 h and 24 h–7 days of age were identified, and the viability classes were redefined, with a narrower class of moderately distressed puppy specific for each BBS. In conclusion, the refining of the AS in dog species is imperative, with cutoff values and viability classifications that must be adapted to the BBS.

## 1. Introduction

Knowledge on canine neonatology has been stagnant for some decades, but has more recently received scientific interest driven by breeders’ quest for reproductive success and veterinarians willing to concede. In contrast to other domestic species, rates of dog perinatal losses are very high, reaching values as high as 20–40%, including 11% of stillbirth and 8–31% of neonatal death, within 3 weeks of age, considered the neonatal period [1,2,3,4,5,6]. High postnatal deaths can be ascribed to the birthing process [7] and intrinsic factors relating to the individual puppies, such as maturity and viability. The process of birth is, in fact, a challenge for all mammals, leading to a quick transition from the well-protected intra-uterine to the challenging extra-uterine environment. The success of this delicate process is facilitated by the finely orchestrated multiorgan changes occurring during the fetal-to-neonatal transition, known as neonatal adaptation. This process is less efficient in altricial species like the dog, in comparison, for example, to species like horses or bovines [8]. Beside fetal maturity, the corner stone for a successful neonatal adaptation is newborn viability, which is defined as the physiologic condition and reactivity of a neonate influencing its chance of surviving. Moreover, the long process of parturition in the dog could be, likely other species, complicated by the occurrence of dystocia, particularly prevalent in some canine breeds, such as small-sized breeds [9].

Having considered all these aspects, a specialized neonatal assistance at birth is needed in dogs as in humans and other domestic species. Neonatal assistance at birth implies a full knowledge about newborn puppy physiology and the availability of useful tools to identify newborns needing special surveillance/assistance or resuscitation. As in other species, considerable progress has also been made in dogs regarding basic knowledge of neonatal physiology in the last years, and several methods for neonatal assessment have been developed starting from the basic evaluation of some behavioral parameters [10,11], some typical neonatal reflexes [12], the canine-adapted Apgar scoring systems [11,13] and also some hematologic evaluations [12,14,15,16,17,18,19]. Blood-gas analysis allows a diagnosis of neonatal asphyxia and acid-base status and is possible with a small amount of blood thanks to the use of portable instruments [12,20]. However, in a clinical setting, a simple, practical method to assess neonate viability is required in order to provide appropriate assistance to those that need it.

The Apgar score, while initially developed in humans, has become a commonly used tool worldwide to assess neonatal vigor under a variety of clinical conditions. The main strengths of the Apgar score are its practical application, quick assessment, lack of need for sophisticated equipment, ease of performance by non-specialist staff and ability to reliably prognosticate survival rates [21]. However, after 50 years of Apgar-score use for the identification of human newborn at risk for mortality, in 2004 Chong and Karlberg [22] refined the Apgar-score cutoff for newborns at risk on the basis of the receiver-operating-characteristics (ROC) statistical analysis, allowing the clinicians and researchers to provide tailored neonatal care rather than following the historical cutoff values.

In dogs as well, the Apgar score has proved its ease of use and many studies have demonstrated its usefulness for the viability evaluation of newborn puppies at birth and for short-term survival prognosis [6,11,13,20,23,24,25,26]. Therefore, it has been considered a useful tool for the routine evaluation of puppies at birth by veterinarians and also by breeders. However, after the first scoring system proposed for dogs in 2009 by Veronesi and co-authors [11], many studies have used the same scoring system for the evaluation of newborn dogs with different measurement timings, suggesting different cutoff values to discern between puppies’ likelihood of survival and puppies that will die [6,13,25]. In addition, one study [13] suggested a small adjustment of the former scoring system for viability assessment at birth in French and English bulldogs, while a study in Chihuahuas confirmed that no changes were needed to the former scoring system for the viability assessment in Chihuahua newborns [26]. However, because of the wide differences among the hundreds of recognized canine breeds, not only with regard to different body size, ranging from the smallest Chihuahua to the biggest Saint Bernard, the possible effect of breed on Apgar scoring should be better investigated in newborn puppies as suggested for other parameters [27,28,29].

Therefore, although it was proved that the Apgar score is an easy, useful tool for routine viability evaluation at birth in newborn dogs, a reevaluation is required to better define some questions raised by its wide use in canine neonatology. The most important questions related to the possible adjustment of the former scoring system refer to: (1) the need for a refining/confirming of the cutoff values for survival, and consequently, for the refining/confirming of newborn viability classes; (2) the need to establish breed-specific cutoff values for survival and lastly (3) the redefinition of newborn viability classes.

For the above-mentioned reasons, the aim of the present study, performed on a retrospective collection of a large number of cases, was the reevaluation of data in surviving and non-surviving puppies and the possible refining of newborn Apgar score cutoff values and viability classes. Moreover, a possible breed-oriented reevaluation of the Apgar score cutoff values between surviving and non-surviving puppies was considered.

## 2. Materials and Methods

### 2.1. Ethical Statement

The study was performed in accordance with the animal welfare committee ethical guidelines and all procedures were carried according to the Italian legislation on animal care (DL 116, 27 January 1992) and the European Guidelines on Animal Welfare (Directive 2010/63/EU). Written informed consent was obtained from each owner to submit pregnant dogs to an elective Caesarean section, for all the necessary clinical procedures on bitches and puppies, and to use clinical records for research purposes.

### 2.2. Animals

The study was performed on data retrospectively collected during a six-year period (2016–2021) in a single veterinary clinic, always by the same surgical (AR, MP) and neonatology (MCV, JF, sometimes MP) team.

All data belong to elective Caesarean section (ECS) cases. The possible reasons for performing ECS were: (a) the bitch belonged to breeds at high risk for dystocia; (b) litter size = 1 or >9; (c) previous history of troubles at parturition.

The protocol of bitches’ breeding, pregnancy diagnosis and monitoring, and ECS scheduling and performing was the same in all the enrolled cases and was the same as previously reported [27]. Specifically, for ECS scheduling, in the last days before the expected date of parturition, mothers and fetuses were submitted to clinical and ultrasonographic daily monitoring for wellbeing and for approaching parturition. In particular, fetal maturity was assessed by gastrointestinal motility detection, as it seems to be a useful parameter for fetal maturity estimation during the last week of pregnancy [30]. Only data from healthy purebred bitches with a normal course of pregnancy were included. All the bitches showed a pre-pregnancy body condition score of 3/5. Data about maternal breed, age and parity, and litter size were recorded.

### 2.3. Newborn Puppies’ Evaluation and Management

At the time of ECS, the following data about newborn puppies were recorded: total number of puppies born; number of stillborn puppies (natimortality); number of newborn puppies affected by severe physical defects or malformations and therefore humanely euthanized; number of newborn puppies born alive that died within the first 24 hours (h) of age (mortality < 24 h); number of puppies alive at 24 h of age that died within 7 days (d) of age (mortality 24 h–7 d); the total number of puppies lost from birth to 7 d of age (total mortality).

All the live-born puppies without physical defects or malformations were individually identified through the application of colored strings around the neck and submitted to viability evaluation through Apgar score measurement. The Apgar score measurement was performed in all cases within 5 min of delivery, according to the system proposed by Veronesi and coauthors [11] and classified in the following viability classes: 0–3 severely distressed, 4–6 moderately distressed, 7–10 not distressed newborns. According to those authors [11], newborns received a different degree of assistance according to their viability classification. Newborns scored as 7–10 were forwarded to routine neonatal management including fluid removal from the upper airways, skin drying, heating and birthweight measurement, and they were put in an incubator until their mothers recovered from anesthesia. Newborns scored from 0 to 6 received a different degree of assistance/resuscitation according to the degree of distress. This tailored assistance starts with manual breathing, stimulation by thorax rubbing and oxygen administration by face mask when needed. Newborns with no heartbeat underwent trans-thoracic cardiac compression and epinephrine (0.01 mg/kg Adrenalina Salf^®^, Salf S.p.A. Laboratorio Farmacologico, Bergamo, Italy) administration. Before discharge, all the newborns received a single dose of high-energy oral solution (Energy Booster^®^, Ozopet, Mantua, Italy) at a dose of 0.5–1 mL/puppy, according to the breed body size.

### 2.4. Statistical Analysis

The statistical analysis was performed using Jamovi 2.3.9.0. The homoscedasticity of the data was firstly assessed by a Levene test. Furthermore, the normality of data distribution was tested using the Shapiro–Wilk test, and because the data were non-normally distributed, the non-parametric Kruskal–Wallis test followed by Dwass–Steel–Critchlow–Fligner pairwise comparisons were used to assess possible differences between breed body sizes (BBSs) regarding the age and parity of the bitch and litter size and newborn birthweight between males and females within each BBS.

The chi-square test was used to assess possible differences in the distribution of females and males within each BBS. The chi-square test was also used to assess possible differences in the distribution of natimortality, mortality < 24 h, mortality 24 h–7 d and total mortality between BBSs and also between males and females within each BBS.

The proportion comparison test was used to assess possible differences in the distribution of newborns in the viability classes according to BBS.

Binomial logistic regression was used to assess possible difference among surviving and non-surviving puppies at <24 h and at 24 h–7 d of age according to newborn gender, Apgar score, viability class and BBS.

The Kruskal–Wallis test followed by Dwass–Steel–Critchlow–Fligner pairwise comparisons were used to assess possible differences in Apgar score among BBSs.

The detection of the Apgar score cutoff value for survival at 24 h and 7 d of age within BBS was calculated by non-parametric Youden ROC analysis, whilst the cutoff for non-survival at 24 h and 7 d of age within BBS was calculated by a non-parametric false positive rate test. Significance was set for *p* ≤ 0.05.

## 3. Results

Data were drawn from 234 litters obtained by the same number of bitches submitted to ECS. The enrolled bitches belonged to 37 canine breeds with diverse distribution, as indicated in Table 1.

Therefore, because of the wide distribution according to the breed, bitches were grouped according to BBS and classified as follows: small (BBS ≤ 10 kg; mean ± SD and min-max: 5.8 ± 2.61, 2.5–9.8 kg), medium (BBS 11–20 kg; mean ± SD and min-max: 14.9 ± 2.09, 11–19 kg) and large (BBS > 20 kg; mean ± SD and min-max: 34.1 ± 10.80, 21–63 kg) [11]. Data about age, parity, litter size and newborn birthweight in the 234 enrolled litters grouped according to BBS are summarized in Table 2.

The statistical analysis showed that age was higher in medium-sized than small- and large-sized bitches, whilst parity was lower in medium- than small- and large-sized bitches. As expected, the smallest litter size was observed in small-sized bitches and the largest in large-sized bitches, as well as the newborn birthweight.

Data about the total puppies born (total number, males and females), and about newborn birthweight, with puppies grouped according to BBS, are reported in Table 3.

The statistical analysis did not show significant differences in the distribution of females and males within each BBS and also the absence of significant differences in the birthweight between males and females within each BBS.

Data about natimortality (17 puppies; 1.6%) plus the four (0.4%) euthanized puppies (two belonging to small- and two to large-sized bitches), neonatal mortality at <24 h after birth and at 24 h–7 d of age and total mortality in puppies grouped according to BBS are reported in Table 4.

The statistical analysis showed the absence of significant differences in the distribution of natimortality, mortality at <24 h, mortality at 24 h–7 d and total mortality among BBS and also between males and females within each BBS.

### 3.1. APGAR

#### 3.1.1. Apgar and Viability Class Distribution in the Total Live-Born Puppies

**Figure 1:**
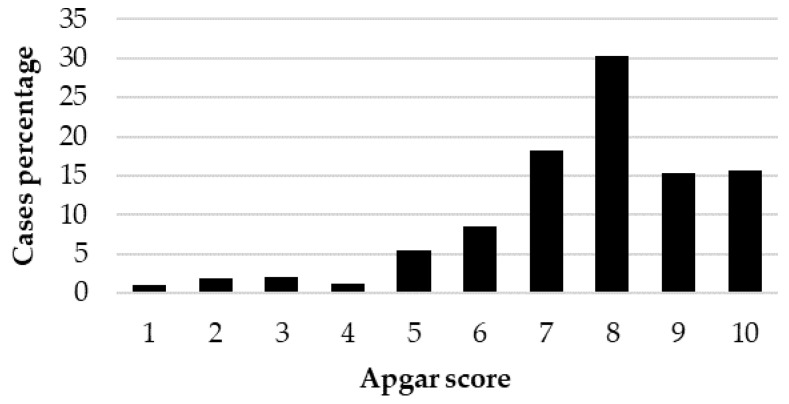
Distribution of the Apgar score (case percentage) in the 1039 live-born puppies.

The distribution of the Apgar score (case percentage) in the 1039 live-born puppies is reported in Figure 1.

The distribution of the 1039 live-born puppies in the viability classes (case percentage) is reported in Table 5.

#### 3.1.2. Apgar and Viability Class Distribution According to Breed Body Size

**Figure 2:**
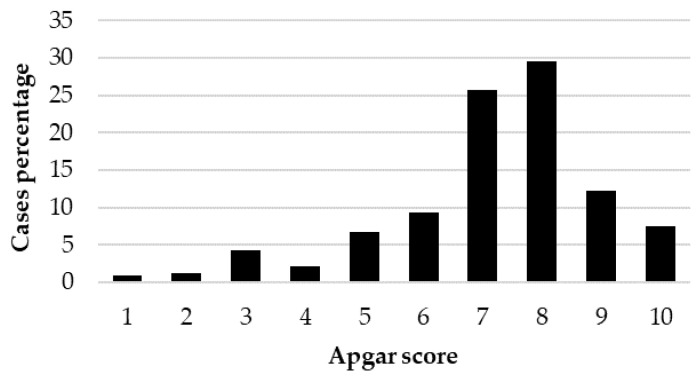
Distribution of the Apgar score (case percentage) in the 412 small-sized live-born puppies.

**Figure 3:**
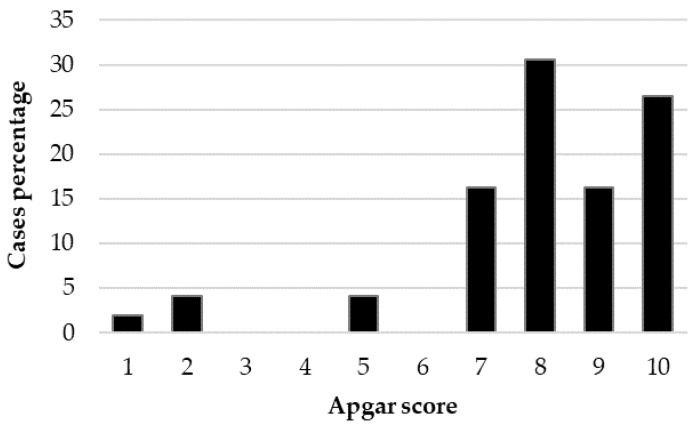
Distribution of the Apgar score (case percentage) in the 49 medium-sized live-born puppies.

**Figure 4:**
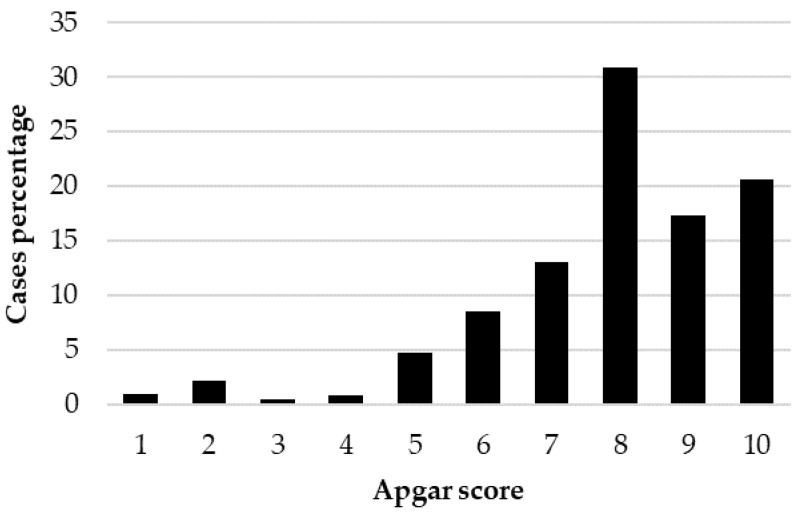
Distribution of the Apgar score (case percentage) in the 578 large-sized live-born puppies.

The Kruskal–Wallis test showed a significant effect of BBS on the Apgar score (*p* < 0.001), with significant differences between large- and small-sized puppies (*p* < 0.001) and between medium- and small-sized puppies (*p* < 0.001). The distribution of Apgar score (case percentage) within each BBS is reported in Figure 2, Figure 3 and Figure 4.

The distribution, expressed as a number and (%), of the 1039 live-born puppies in the viability classes according to the BBS is reported in Table 6.

The proportion comparison test showed a significantly (*p* < 0.05) higher proportion of small-sized than large-sized puppies in the viability class 0–3. In the viability class 4–6, a higher proportion of small- than medium-sized (*p* < 0.05) and of large- than medium-sized puppies (*p* < 0.05) were found. In the viability class 7–10, a higher proportion of medium- than small-sized puppies (*p* < 0.05) and of large- than small-sized puppies (*p* < 0.01) was observed.

#### 3.1.3. Apgar Score and Survival at 24 h and at 7 Days of Age

The bimodal logistic regression showed the effect of the Apgar score on the “chance” (odds ratio and confidence interval, OR (95% CI)) of a puppy’s survival. Every point by which the Apgar score increased significantly improved the puppy’s chance of survival at 24 h (OR (95% CI): 3.42 (2.38–4.89), *p* < 0.001) and at 7 d of age (OR (95% CI): 2 (1.61–2.49), *p* < 0.001).

#### 3.1.4. Viability Class and Survival at 24 h and at 7 Days of Age

The distribution expressed as number and (%) of newborn survival at 24 h and at 7 d of age according to viability classification is reported in Table 7.

The bimodal logistic regression applied to the viability class showed an effect of the viability classes on the “chance” of a puppy’s survival at 24 h and at 7 d of age. In detail, in puppies classified as not distressed (Apgar score 7–10), the chance of survival at 24 h of age was significantly higher (OR (95% CI): 368.88 (66.95–2032.52), *p* < 0.001) than puppies included in the severe-distress class (Apgar score 0–3) of viability. However, puppies classified in the moderate-distress class (Apgar score 4–6) also had a higher chance (OR (95% CI): 63.94 (13.95–293.14), *p* < 0.001) of survival at 24 h of age in comparison to puppies included in the severe-distress class (Apgar score 0–3).

Furthermore, at 7 d of age the chance of a puppy’s survival was significantly higher (OR (95% CI): 63.49 (19.15–210.46) *p* < 0.001;) in puppies classified as not distressed (Apgar score 7–10) than severely distressed puppies (Apgar score 0–3), but again also in moderately distressed puppies (Apgar score 4–6) the chance of survival was higher (OR (95% CI): 20.05 (5.35–75.09) *p* < 0.001) than in severely distressed (Apgar score 0–3) newborns.

The distribution, expressed as a number and (%) of newborn survival at 24 h and at 7 d of age, according to viability classification and the BBS, is reported in Table 8.

The bimodal logistic regression showed an effect played by BBS on the chance of puppy’s survival at 24 h of age in the 0–3 viability class, with severely distressed small-sized puppies having a higher chance of survival (OR (95% CI): 20.95 (2.48–176.93), *p* < 0.01) as compared to severely distressed large-sized puppies.

#### 3.1.5. Detection of the Apgar Score Cutoff Value for Survival within Breed Body Size

Because of the demonstrated effect of BBS on puppies’ viability and on Apgar score, the non-parametric Youden test was used to calculate the ROCs for the definition of the Apgar-score cutoff values for survival at 24 h of age and at 7 d of age for each one of the three BBSs (small, medium, large). The Apgar cutoff values for survival at 24 h and at 7 d of age according to BBS (small, medium, large), with the positive predictive value (PPV), negative predictive value (NPV), sensitivity and specificity, are reported in Table 9.

#### 3.1.6. Detection of the Apgar Cutoff Value for Death at <24 h and at 24 h–7 Days of Age

According to the ROC calculated through the false positive rate, the cutoff value for death at <24 h and at 24 h–7 d of age was 3 for both small- and large-sized puppies, with a death rate >99%. Unfortunately, due to the limited number of dead medium-sized puppies, the cutoff value for death in this BBS was not calculated. However, because all the medium-sized puppies Apgar scored as 0–3 did not survive, the cutoff value of 3 for death risk at <24 h of age is merely cautiously suggested.

#### 3.1.7. Redefinition of Viability Classes According to Detected Apgar Score Cutoff Values

On the basis of the newly detected cutoff values for surviving and for death at <24 h and 24 h–7 days of age, the viability classes have been redefined according to BBS as reported in Table 10.

## 4. Discussion

This study, based on the retrospective analysis of a large number of cases, aimed to assess the need for a redefinition of Apgar score cutoff values for survival and non-survival prognosis and consequently the possible redefinition of the viability classes.

### 4.1. Canine Breed and Breed Body Size

Because data were drawn only from purebred bitches, the first consideration relates to the choice of grouping the many breeds enrolled according to breed body-size classes. Although a breed-specific reevaluation of Apgar scoring should be the final goal, the wide distribution of the enrolled bitches in over 30 breeds prevented this approach, so that the body-size grouping was chosen. Unfortunately, despite the suitable number of litters in the small (55%) and large (45%) BBS, the medium-sized group was very poorly represented (5%). Underrepresentation of medium-sized breeds could have been related to people’s preferences. People living in a large city such as Milan strongly prefer small-sized dogs, whilst people living in the countryside around Milan prefer large-sized breeds. Because of the underrepresented number of medium-sized puppies, the results related to the medium-sized group will not be included in the discussion, which will be limited to the small and large BBS.

### 4.2. Maternal Data

The vast majority of the enrolled bitches were in the optimal age for breeding. Data about age and parity were very similar to those previously reported [23]. As expected, in agreement with other authors [31] a significant difference in litter size was observed among the BBSs, with the highest number of puppies in large-sized litters and the smallest in the small-sized, in agreement with Borge et al. [32].

### 4.3. Newborn Mortality

The percentage of losses because of stillbirth plus euthanasia for severe malformations, mortality within 24 h, mortality 24 h–7 d of age and total mortality, were 2%, 3.5%, 3.2% and 8.4%, respectively. Although a detailed meta-analysis of literature is not possible due to the different parameters considered in those studies, the 2% natimortality plus euthanasia is lower than the 6.4–14% reported in other studies [7,11], respectively. The 5.5% losses occurring within the first 24 h of age are higher than the 0.66-3.4% previously reported [6,7,13,30] but very similar to the 5.4% reported by Veronesi et al. [11] and lower than the 11.8% reported by Munnich and Kuchenmeister [5]. The 8.4% of total losses within 7 d of age are a bit lower than the 12.4–15% reported in literature by [33,34], respectively. Interestingly, no significant differences in the distribution of natimortality, mortality within 24 h, mortality 24 h–7 d of age and total mortality among BBS and also between males and females within each BBS were found, in agreement with Mila et al. [31]. When differences between males and females’ birthweight within each BBS was assessed, no significant differences were found, as previously reported [31].

### 4.4. Newborn Distribution According to Apgar Score

Focusing on Apgar score, the distribution of the 1039 puppies born alive showed that 80% were classified as not distressed puppies, 15% as moderately distressed puppies and 5% as severely distressed puppies. These results are very similar to the 85.3, 10.4 and 4.3%, respectively, previously reported [11], but consistently different from data reported by Batista et al. [13] (33.1, 46.3 and 20.5%, respectively), Titkova et al. [7] (51.4, 5.4, 43.2%, respectively) and by Doebeli et al. [23] (68, 15 and 17% with alfaxalone induction and 19, 31 and 50% with propofol induction). The strong differences among studies could be, however, attributable to different study designs, including canine breeds, type of parturition, different anesthetic protocols and different assessment times for the Apgar score. When grouped according to BBS, the statistical analysis showed a significant effect of BBS on the Apgar score with significant differences between large- and small-sized puppies, highlighting the effect of breed-size physiologic peculiarities on newborn viability.

### 4.5. Newborn Distribution According to Viability Class

Concerning the viability classification according to BBS, the small-sized newborns showed a lower percentage of not distressed puppies in comparison to large-sized newborns (75 and 82%, respectively); in the moderate-distress class a similar percentage of puppies were included as for small- and large-sized puppies (18 and 14%, respectively). A higher percentage was found in the small-sized category in comparison to large-sized newborns (6.5 vs. 3.7%). This 6.5% of severely distressed neonates in the small BBS is in agreement with the 10% previously reported [26] in Chihuahua dogs and seems to further support the hypothesis of a possible major vulnerability of small-sized puppies at the time of birth.

### 4.6. Apgar Score and Viability Class and Survival at 24 h and at 7 Days of Age

When the Apgar score was considered in relation to survival at 24 h and at 7 d of age, the statistical analysis showed a significant effect played by increasing Apgar score on the chance of survival. Although expected, this result highlighted that the Apgar score remains a reliable tool for the prognosis of survival in newborn dogs. According to viability classes in relation to survival at 24 h and 7 d of age, 99%, 95% and 59% of puppies scored as 7–10, 4–6 and 0–3, respectively, were alive at 24 h of age. The 99% survival in puppies scored as 7–10 was in agreement with previous studies reporting 95–100% survival [7,11,25,26,35,36,37]. Similarly, the 95% survival of puppies scored as 4–6 was superimposable on the 94–100% previously reported [7,25,26]. The 0–3 score remains confirmed to be for those newborns with the worst chance for survival [11,13,25], as also reported in humans [22]. Interestingly, both Apgar score and viability class showed an effect on the chance of survival at 7 d of age; previously, Veronesi et al. [11] reported that the Apgar score should be considered only for short-term survival prognosis, as reported for human babies [22]. On the other hand, Mila et al. [6] found that the Apgar score was predictive of newborn dogs’ mortality within 21 days of age, similar to data reported in human babies for the neonatal period (up to 28 days of age) [21].

### 4.7. BBS and Survival at 24 h and at 7 Days

Note that the statistical analysis showed an effect played by BBS on the chance of a puppy’s survival at 24 h of age in the 0–3 viability class, with severely distressed small-sized puppies having a higher chance of survival as compared to severely distressed large-sized puppies. This finding seems to suggest that, although more small-sized newborns risk being classified as severely distressed, when appropriate assistance is provided, they have a better chance to survive in comparison to severely distressed large-sized newborns.

### 4.8. Refining the Cutoff for Survival and Death at 24 h and at 7 Days of Age within Breed Body Size

Because of the significant effect of BBS on Apgar score distribution and also on survival at 24 h and 7 d of age, the BBS was taken in consideration for the refining of the best Apgar score cutoff value for survival and for death within 24 h and 24 h–7 d of age. Reliable Apgar score cutoff values for survival and for death were refined for small- and for large-sized newborns through ROCs. Focusing on survival prognosis at 24 h of age, some comments can be addressed to the Apgar score cutoff value detected in the present study, which differs from the cutoff value formerly proposed [11]. In the present study, the Apgar score cutoff value for survival was 5 for small-sized and 6 for large-sized puppies in comparison to the former “general” Apgar score cutoff value of 7. This means that a number of puppies previously considered moderately distressed should actually be considered as not distressed newborns. This observation is consistent with clinicians’ perception that puppies scored 4 to 6 and consequently classified as moderately distressed were in fact very uneven, with puppies scored 6 very similar to not distressed ones and with puppies scored 4 very close to the severely distressed class of viability. The new cutoff values reported in the present study are in agreement with authors suggesting a cutoff value of ≥4 [13] or 5 [25]. When compared to one study in human babies [22], we note that those authors suggested a 5-min Apgar score cutoff value of >8 to consider healthy infants.

When survival prognosis is considered for puppies of 7 d of age, the cutoff values did not change for small-sized but increased to 8 in large-sized puppies. This result should, however, be considered cautiously. In fact, although a significant effect is exerted by Apgar scoring on survival at 7 d of age, it has to be considered that the many causes of puppy mortality in the first week may not at all be related to puppy viability at birth. This could explain why at 7 d of age the survival cutoff was raised to 8 in large-sized puppies, as evidenced also by the decreased sensibility and specificity. Indeed, in the authors’ experience, puppies’ death in large-sized breeds can be caused by mother crushing more frequently than in small-sized mothers, mainly due to clumsy movements of the dam, and this could also concern puppies that showed normal viability at birth. A median Apgar score cutoff value of 6 was reported for predicting mortality in newborn puppies at 1 day of age [6]. The same cutoff value was also suggested for predicting mortality at 21 days of age, although a poor likelihood of predictive mortality during the neonatal period was found. Additionally, Chong and Karlberg [22] confirmed the effectiveness of the Apgar scoring system in humans for the assessment of vitality in newborns and for predicting survival in the period of time immediately after birth.

In contrast, as far as the prognosis of death was concerned at 24 h and 7 d of age, the Apgar score cutoff value was similar in all the BBSs and was in agreement with the former cutoff value proposed by Veronesi et al. [11], but also with the value of 3 suggested by other authors [20,26] as the threshold for an increased risk of death. The present study cutoff is different from the value of 6 proposed by Mila et al. [6], obtained, however, in a study in which the Apgar measurement was performed at a variable time after birth and therefore had a different scheduling when compared to the present study. The risk of death in severely distressed newborns despite assistance/resuscitation was as high as 41%, in agreement with the range of 0–41% reported by some studies [7,20,24,25,35,37] and somewhat lower than the 61% reported by Fusi et al. [26]. Additionally, Chong and Karlberg [22] confirmed that an Apgar score 0–3 in human babies was associated with death at up to 2 days of age, in agreement with a previous report [21].

### 4.9. Refining of Viability Classes

On the basis of newly detected cutoff values for survival and death at 24 h and at 7 d of age, the viability classes were accordingly depicted within each BBS. Severely distressed class did not change, with puppies Apgar scored from 0 to 3 considered “critical” newborns with the worst chance of survival within 24 h and 7 d of age. Interestingly, the moderately distressed class was defined differently in comparison to the system formerly proposed [11]. In the present study, in fact, the moderately distressed class should encompass puppies scored 4 in small-sized dogs and 4–5 in (medium-sized and) large-sized dogs at 24 h of age. From a clinical standpoint, this represents the most important class of newborns. Indeed, the not distressed puppies do not need special assistance, but only routine neonatal management as suggested for human babies, while severely distressed puppies have a higher chance of dying despite neonatal assistance or resuscitation. On the other hand, the moderately distressed class includes puppies that can survive thanks to appropriate assistance, but need to be identified in time.

Lastly, the not distressed class was widened in comparison to the former one [11] in all the BBS. In fact, in small-sized puppies this class included newborns scored from 5 to 10 for survival prognosis at 24 h and at 7 d of age. In large-sized puppies, those scored from 6 to 10 were included in the not distressed class for prognosis at 24 h of age and from 8 to 10 for prognosis at 7 d of age. This finding is important from a clinical standpoint, because it suggests that according to the past scoring system, a certain number of not distressed puppies were incorrectly considered moderately distressed, wasting unnecessary neonatal assistance that can now be better dedicated to newborns actually requiring special care.

## 5. Conclusions

In conclusion, the results of the present study showed that the Apgar score evaluation of newborn dogs, still reliable for identifying puppies needing assistance/resuscitation and for short-term survival prognosis, must consider the differences related to breed body-size characteristics, with cutoff values and viability classifications adapted to the specific BBS.

Moreover, in comparison to previous studies, a narrow class of moderately distressed puppies was defined, especially for small-sized breeds. Even if the newborns classified in this class represent the most crucial neonates for the clinician, their correct detection followed by timely and appropriate assistance could improve their chance of survival. Lastly, small-sized puppies are more represented in the severely distressed class but have a better chance of survival in comparison to large-sized newborns.

## Figures and Tables

**Table 1:** Breed distribution (number and %) of the 234 bitches/litters enrolled in the present study.

Breed	Bitches	
	*n*	%
American Staffordshire terrier	1	0.4
Australian cattle dog	5	2.1
French bulldog	9	3.9
Poodle toy	12	5.1
Poodle giant	1	0.4
Dachsund	7	3
Beagle	3	1.3
Border collie	1	0.4
Bernese mountain dog	9	3.9
Boxer	4	1.7
Epagneul breton	2	0.85
American bulldog	1	0.4
English bulldog	14	6
Pug	5	2.1
Chihuahua	21	9
American cocker spaniel	1	0.4
English cocker spaniel	1	0.4
Dobermann	8	3.4
Golden retriever	1	0.4
Hovawart	2	0.85
Irish wolfhound	6	2.6
Jack russell terrier	8	3.4
Labrador retriever	2	0.85
Leonberger	7	3
Maltese	27	11.5
Maremma sheepdog	14	6
Bull terrier miniature	16	6.8
Pekingese	1	0.4
German shepherd	15	6.4
Rottweiler	3	1.3
Saint Bernard	3	1.3
English setter	3	1.3
Shar-pei	2	0.85
Shihtzu	2	0.85
Spitz	13	5.6
Newfoundland	2	0.85
Yorkshire terrier	2	0.85

**Table 2:** Data about age, parity, litter size and birthweight in the 234 litters grouped according to BBS (small, medium, large). Data are expressed as median (min–max).

	Small	Medium	Large
**Number (%) of litters**	123 (52.6)	12 (5.1)	99 (42.3)
**Age (years): median** **(min–max)**	4 ^a^ (1.5–9)	5 ^b^ (2–10)	4 ^a,c^ (1.5–9)
**Parity: median** **(min–max)**	2 ^a^ (1–7)	1 ^b^ (1–4)	2 ^a,c^ (1–5)
**Litter size (*n*): median** **(min–max)**	4 ^a^ (1–7)	6 ^b^ (1–8)	7 ^c^ (1–15)
**Birthweight (g): median** **(min–max)**	160 ^a^ (60–390)	318 ^b^ (190–520)	500 ^c^ (217–930)

^a,b,c^ denote within-row significant differences with *p* < 0.001.

**Table 3:** Data about the total born puppies (total number, males and females), and about birthweight, with puppies grouped according to BBS. Data regarding birthweight are expressed as a median (min–max).

	Total Born Puppies (%)			Birthweight (g)		
	Males	Females	Total	Males	Females	Total
**Small**	210 (49.6%)	213 (50.4%)	423/1060 (39.9%)	160 (60–390)	150 (60–330)	160 (60–390)
**Medium**	27 (54%)	23 (46%)	50/1060 (4.7%)	280 (190–460)	348 (200–520)	318 (190–520)
**Large**	302 (51.4%)	285 (48.6%)	587/1060 (55.4%)	520 (260–930)	490 (217–900)	500 (217–930)
**Total**	539 (50.8%)	521 (49.2%)	1060/1060 (100%)			

**Table 4:** Data about the natimortality plus euthanized puppies, neonatal mortality at < 24 h after birth and at 24 h–7 days (d) of age, and total mortality in puppies grouped according to BBS.

	Natimortality + Euthanized			Mortality < 24 h			Mortality 24 h–7 d			Total Mortality
	Males	Females	Total	Males	Females	Total	Males	Females	Total	
**Small**	6	5	11/423 (2.6%)	10	5	15/412 (3.6%)	7	8	15/397 (3.8%)	41/423 (9.7%)
**Medium**	1	-	1/50 (2%)	-	2	2/49 (4.1%)	-	1	1/47 (2.1%)	4/50 (8%)
**Large**	6	3	9/587 (1.5%)	6	13	19/578 (3.3%)	7	9	16/559 (2.9%)	44/587 (7.5%)
**Total**	13	8	21/1060 (2%)	16	20	36/1039 (3.5%)	14	18	32/1003 (3.2%)	89/1060 (8.4%)

**Table 5:** Distribution (expressed as number and %) of the 1039 live-born puppies in the viability classes.

Viability Class	Puppies *n* (%)
**0–3**	51 (4.9%)
**4–6**	160 (15.4%)
**7–10**	828 (79.7%)
**Total**	1039 (100%)

**Table 6:** Distribution (expressed as number and %) of the 1039 live-born puppies in the viability classes according to the BBS.

	Small	Medium	Large
**0–3**	27 (6.5%) ^a^	3 (6.1%) ^a,b^	21 (3.7%) ^b^
**4–6**	76 (18.4%) ^a^	2 (4.1%) ^b^	82 (14.2%) ^a,c^
**7–10**	309 (75.1%) ^a^	44 (89.8%) ^b^	475 (82.1%) ^b,d^
**Total**	412 (100%)	49 (100%)	578 (100%)

^a,b^ and ^a,c^ denote within-row significant differences with *p* < 0.05; ^b,d^ denotes within-row significant differences with *p* < 0.01.

**Table 7:** Distribution (expressed as number and %) of newborn survival at 24 h and at 7 days (d) of age according to viability classification.

	Viability Class			Total
	0–3	4–6	7–10	
**Alive at 24 h** ***n* (%)**	30/51 (58.8%)	152/160 (95%)	821/828 (99.2%)	1003/1039 (96.5%)
**Alive at 7 d** ***n* (%)**	21/30 (70%)	145/152 (95.4%)	805/821 (98.1%)	971/1003 (96.8%)

**Table 8:** Distribution (expressed as number and %) of newborn survival at 24 h and at 7 days (d) of age according to viability classification and to the BBS.

	Viability Class					
	0–3		4–6		7–10	
	Alive 24 h *n* (%)	Alive 7 d *n* (%)	Alive 24 h *n* (%)	Alive 7 d *n* (%)	Alive 24 h *n* (%)	Alive 7 d *n* (%)
**Small**	24/27 (88.9%) ^a^	18/24 (75%)	70/76 (92%)	68/70 (97.1%)	305/309 (98.7%)	297/305 (97.4%)
**Medium**	0/3 (0%)	0	2/2 (100%)	2/2 (100%)	43/44 (97.7%)	43/43 (100%)
**Large**	6/21 (28.6%) ^b^	3/6 (50%)	80/82 (97.6%)	75/80 (93.8%)	473/475 (99.6%)	465/473 (98.3%)
**Total**	30/51 (58.8%)	21/30 (70%)	152/160 (95%)	145/152 (95.4%)	821/828 (99.2%)	805/821 (98.1%)

^a,b^ denotes within column significant difference for *p* < 0.01.

**Table 9:** Apgar cutoff values for survival at 24 h and 7 days (d) of age according to BBS (small, medium, large), with the positive predictive value (PPV), negative predictive value (NPV), sensitivity and specificity.

	Apgar Score 24 h/7 d	PPV 24 h/7 d	NPV 24 h/7 d	Sensitivity 24 h/7 d	Specificity 24 h/7 d
**Small**	5/5	0.989/0.976	0.306/0.280	0.937/0.953	0.733/0.438
Medium *	6*/4**				
**Large**	6/8	0.998/0.987	0.352/0.069	0.938/0.727	0.950/0.688

* In medium-sized puppies, the limited number of puppies prevented the actual calculation of the cutoff values for survival at 24 h and 7 d of age. Therefore, the estimated cutoff value for survival at 24 h of age was suggested to be 6, on the basis of the lack of deaths at that Apgar score value. ** At 7 d of age, because all the medium-sized puppies were alive, a cutoff value for survival could not be determined. Merely for the sake of completeness, all the puppies alive at 7 d of age were Apgar scored ≥4.

**Table 10:** Redefinition of viability classes according to detected Apgar-score cutoff values.

	Small			Medium *			Large		
	Severe Distress	Mode Distress	No Distress	Severe Distress	Moderate Distress	No Distress	Severe Distress	Moderate Distress	No Distress
**<24 h**	0–3	4	5–10	0–3	4–5	6–10	0–3	4–5	6–10
**24 h–7 days**	0–3	4	5–10	0–3	4–5	6–10	0–3	4–7	8–10

* In medium-sized puppies the newly detected viability classes were merely cautiously suggested, because they were not calculated by ROC, and data were drawn from a limited number of newborns.

## Data Availability

The data are available upon reasonable request to the corresponding author.
